# Combination of sarcopenia and systemic inflammation-based markers for predicting the prognosis of patients undergoing pancreaticoduodenectomy for pancreatic cancer

**DOI:** 10.1371/journal.pone.0305844

**Published:** 2024-06-24

**Authors:** Masashi Utsumi, Masaru Inagaki, Koji Kitada, Naoyuki Tokunaga, Kosuke Yunoki, Hiroki Okabayashi, Ryosuke Hamano, Hideaki Miyasou, Yousuke Tsunemitsu, Shinya Otsuka

**Affiliations:** Department of Surgery, NHO Fukuyama Medical Center, Fukuyama City, Hiroshima, Japan; School of Medicine, Tottori University, JAPAN

## Abstract

**Background:**

This study aimed to evaluate the effects of sarcopenia and inflammation on the prognosis of patients with pancreatic cancer after pancreaticoduodenectomy.

**Methods:**

Eighty patients who had undergone pancreaticoduodenectomy for pancreatic cancer between July 2010 and December 2023 were included in this study. The psoas muscle index was used to assess sarcopenia. The C-reactive protein-to-albumin ratio, prognostic nutritional index, neutrophil-to-lymphocyte ratio, and platelet-to-lymphocyte ratio were used to calculate the preoperative inflammatory marker levels. The prognostic factors for overall survival were determined using Cox regression analysis.

**Results:**

Twenty-four patients were diagnosed with sarcopenia. Sarcopenia showed a significant association with advanced tumor stage. Univariate analysis revealed a significant reduction in overall survival in patients with a prognostic nutritional index of <45, C-reactive protein-to-albumin ratio of ≥0.047, cancer antigen 19–9 levels of ≥130 U/mL, sarcopenia, lymph node metastasis, and vascular invasion. Multivariate analysis revealed that a C-reactive protein-to-albumin ratio of ≥0.047 (hazards ratio, 3.383; 95% confidence interval: 1.384–8.689; *p*< 0.001), cancer antigen 19–9 levels of ≥130 U/mL (hazards ratio, 2.720; 95% confidence interval: 1.291–6.060; *p* = 0.008), sarcopenia (hazards ratio, 3.256; 95% confidence interval: 1.535–7.072; *p* = 0.002) and vascular invasion (hazards ratio, 2.092; 95% confidence interval: 1.057–4.170; *p* = 0.034) were independent predictors of overall survival. Overall survival in the sarcopenia and high C-reactive protein-to-albumin ratio groups was significantly poorer than that in the non-sarcopenia and low C-reactive protein-to-albumin ratio and sarcopenia or high C-reactive protein-to-albumin ratio groups.

**Conclusion:**

Sarcopenia and a high C-reactive protein-to-albumin ratio are independent prognostic factors in patients with pancreatic cancer after pancreaticoduodenectomy. Thus, sarcopenia may have a better prognostic value when combined with the C-reactive protein-to-albumin ratio.

## Introduction

Pancreatic cancer, one of the most lethal types of cancer, is the seventh leading cause of cancer-related death worldwide [1]. Approximately 50–55% of patients present with metastatic disease at the time of diagnosis, whereas 30–35% present with locally advanced unresectable disease. Only 15–20% of patients present with resectable (R) or borderline resectable (BR) disease [2,3]. Adjuvant chemotherapy improves survival outcomes; however, surgical resection remains the only potentially curative approach [4]. Advances in perioperative diagnosis and management, as well as surgical techniques, have improved the overall survival of patients with pancreatic cancer after resection. Nevertheless, the 5-year post-resection survival rate of pancreatic cancer remains 20–30% [3]. Thus, the identification of reliable and sensitive preoperative prognostic factors is necessary to aid in individualized treatment decision-making and survival outcome prediction for pancreatic cancer.

Pancreaticoduodenectomy is indicated for the resection of cancer involving the pancreatic head. Previous studies have revealed that malnutrition and low preoperative albumin levels are associated with an increased risk of morbidity and mortality among patients undergoing pancreaticoduodenectomy [5]. In addition, inflammatory markers have also been identified as useful prognostic indicators for pancreatic cancer after pancreaticoduodenectomy [6].

Tumor-specific factors, such as tumor size, tumor differentiation, lymph node metastasis, vascular invasion, and resection margin status, influence the prognosis of patients after pancreaticoduodenectomy. However, the postoperative prognosis is multifactorial. Moreover, it is related to tumor-specific factors and patient characteristics. Sarcopenia, a disease characterized by the loss of skeletal muscle mass and strength, has been identified as a factor indicating poor prognosis in patients undergoing digestive surgery [7,8]. As for pancreatic cancer, sarcopenia has been identified as an independent risk factor for overall survival after resection [9,10]. Consequently, the prediction and early diagnosis of sarcopenia play a crucial role in determining the prognosis of patients with pancreatic cancer.

Inflammation and sarcopenia influence carcinogenesis and the progression of cancer [11]. There is increasing evidence that the systemic inflammatory response plays an important role in the progression of various cancers [12]. Previous studies have assessed systemic inflammation-based biomarkers, such as the neutrophil-lymphocyte ratio [13], platelet-lymphocyte ratio [14], prognostic nutritional index [15], and C-reactive protein-to-albumin ratio (CAR) [16]. A correlation has been observed between these biomarkers and the postoperative prognosis in patients with pancreatic cancer [17–21].

Systemic inflammation has been observed in patients with sarcopenia [22]. A correlation has been observed between sarcopenia accompanied by systemic inflammation and poor prognosis in patients with various types of cancer [23–25]. However, no study has examined the effect of sarcopenia in combination with systemic inflammation on the prognosis of patients with pancreatic cancer undergoing pancreaticoduodenectomy. Therefore, this study aimed to evaluate the effects of sarcopenia and inflammation on the postoperative prognosis of patients with pancreatic cancer.

## Methods

### Patients

Eighty consecutive patients with pancreatic cancer who had undergone pancreaticoduodenectomy at the Department of Surgery, National Hospital Organization Fukuyama Medical Center, between July 1, 2010 and December 1, 2023 were included in this retrospective study. Data were collected and analyzed from December 6, 2023 to January 15, 2024 to allow for at least 1 month of follow-up. Diagnosis of pancreatic cancer was confirmed by pathological examination. All procedures involving human participants performed in this study adhered to the ethical standards of the Institutional and National Research Committee and the 1964 Declaration of Helsinki and its later amendments or comparable ethical standards. The Ethics Review Committee of Independent Administrative Agency, National Hospital Organization Fukuyama Medical Center approved this clinical research (approval number: ERB2023033). The requirement for obtaining written informed consent from the patients was waived owing to the retrospective study design.

### Data collection

Data regarding the following clinicopathological characteristics were extracted from the medical records of the participants: demographic characteristics (age at the time of undergoing surgery, sex, and body mass index), preoperative laboratory data (platelet/neutrophil/lymphocyte count and serum albumin, carcinoembryonic antigen, and cancer antigen 19–9 [CA19-9] levels), CAR, platelet-lymphocyte ratio, neutrophil-lymphocyte ratio, prognostic nutritional index, operative blood loss, surgical duration, resectability, tumor size/stage (Union for International Cancer Control Tumor–Node–Metastasis classification [eighth edition]), vascular invasion, and tumor differentiation [26]. CAR was calculated using the following equation [16]:

C-reactive protein-to-albumin ratio = C-reactive protein (mg/dL)/serum albumin (g/dL).

The neutrophil-lymphocyte ratio and platelet-lymphocyte ratio were calculated by dividing the neutrophil and platelet counts by the lymphocyte count, respectively [19,27]. The prognostic nutritional index was calculated using the following equation:

10 × serum albumin (g/dL) + 0.05 × total lymphocyte count (/mm^3^) [20,28].

The Clavien–Dindo classification was used to classify the complications [29]. Postoperative complications were defined as complications of grade ≥3. The International Study Group on Pancreatic Fistula classification was used to diagnose and grade pancreatic fistulas [30,31]. Death within 30 days of surgery was defined as postoperative mortality. The administration of two cycles of neoadjuvant chemotherapy with gemcitabine + S-1 has been recommended for all cases since 2019 [32–34]. Adjuvant chemotherapy with TS-1 was continued for approximately 6 months postoperatively [35].

### Sarcopenia marker

**Fig 1:**
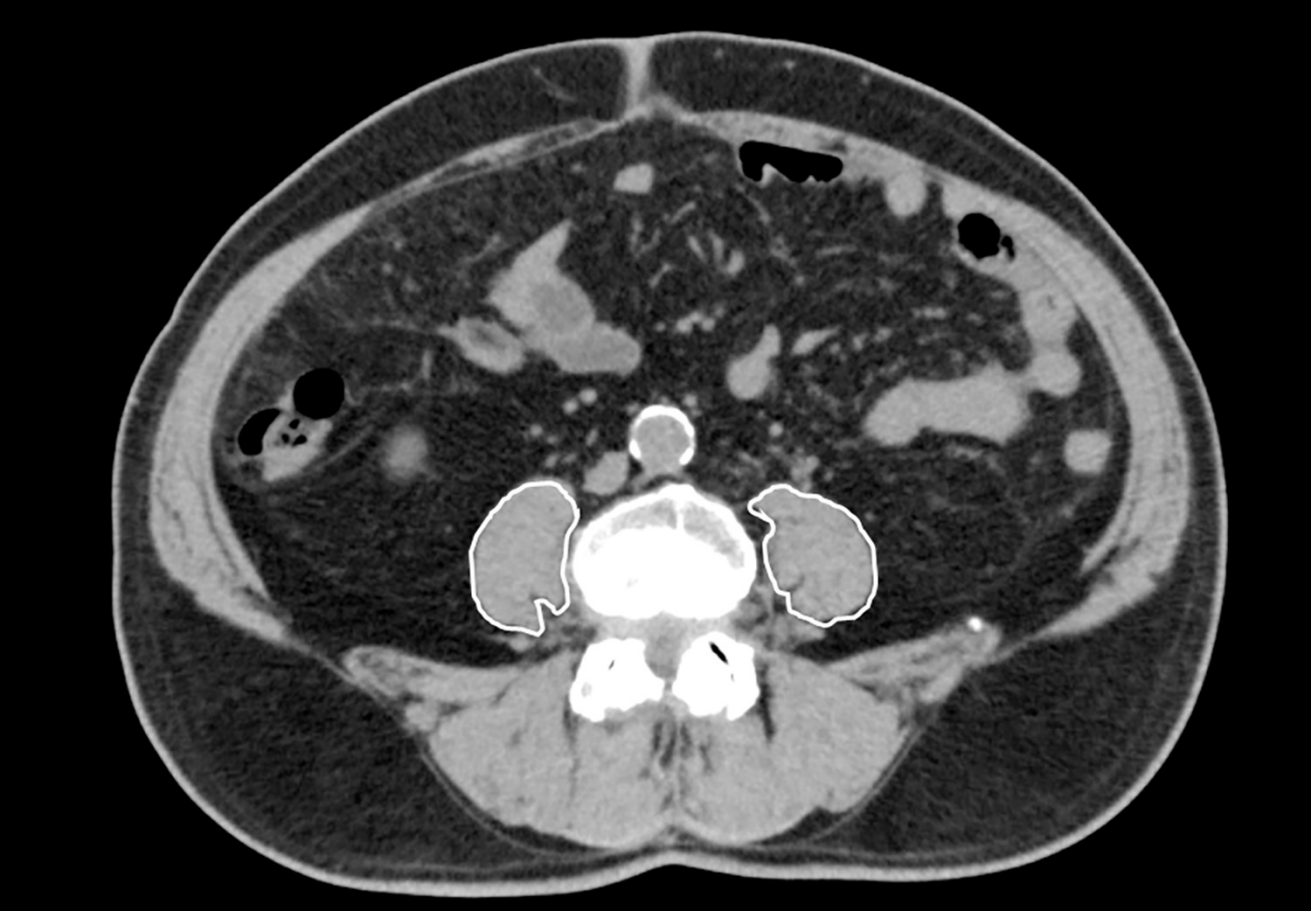
Cross-sectional area of the bilateral psoas muscles at the level of the third lumber vertebra.

The psoas muscle index (PMI), an alternative measurement of sarcopenia adopted by the Japan Society of Hepatology [9], was used to assess sarcopenia. Preoperative computed tomography imaging examinations were conducted within 1 month before the surgery using a multidetector computed tomography scanner (Aquilion CXL 64; Canon Medical Systems, Tochigi, Japan). The cross-sectional area of the bilateral psoas muscles was measured on computed tomography images acquired at the level of the caudal end of the third lumbar vertebra via manual tracing (Fig 1). PMI was calculated using the following formula:

Psoas muscle index = cross-sectional area of the bilateral psoas muscles (cm^2^)/height squared (m^2^).

A low psoas muscle index indicated low muscle volume [9]. The PMI range for women differs from that for men; therefore, different cut-off values were established using receiver operating characteristic (ROC) curves. The optimal cut-off values were selected on the basis of the best accuracy for the outcome. PMI values below the cut-off value indicated sarcopenia.

### Operative procedure

Four surgeons specializing in pancreatic surgery performed subtotal stomach-preserving pancreaticoduodenectomy by an open approach. The extent of locoregional lymphadenectomy was determined on the basis of the preoperative diagnosis of the patient. A modification of the Child method was used for surgical reconstruction [36]. The proximal jejunal stump was passed through the retrocolic pathway. Pancreaticojejunostomy, biliojejunostomy, and gastrojejunostomy were performed subsequently. The modified Kakita (*n* = 21; July 2010 to June 2015) or modified Blumgart (*n* = 59; July 2015 to the present) anastomosis methods were used to perform pancreaticojejunostomy [37]. The decision to insert plastic stents into the main pancreatic duct for internal drainage was made intraoperatively by each surgeon. Two drains were placed posterior to the pancreaticojejunostomy and hepaticojejunostomy anastomoses.

### Follow-up

The patients underwent routine follow-up until December 2023. Data regarding the medical history (symptoms and findings of the physical examination), results of laboratory studies, and imaging findings were collected during the follow-up visits scheduled every 3–6 months for ≥5 years. The duration between surgery and death or the last follow-up was defined as overall survival. The duration between surgery and recurrence was defined as recurrence-free survival.

### Statistical analyses

The data were blinded before analysis and are presented as mean ± standard deviation. Univariate analysis was performed using the chi-squared test. The area under the ROC curve (AUC) was used to determine the diagnostic accuracy. The optimal cut-off value for each parameter was calculated by maximizing the Youden index (sensitivity + specificity − 1) [38]. Overall survival and recurrence-free survival were estimated using the Kaplan–Meier method. The differences between the subgroups were compared using the log-rank test. A Cox proportional hazards model was used to perform univariate and multivariate analyses. Significant variables in univariate analysis were included in multivariate analysis. A *p*-value of <0.05 was considered statistically significant. JMP version 11 (SAS Institute, Cary, NC, USA) was used to perform all statistical analyses.

## Results

### Patient characteristics

Eighty consecutive patients, comprising 40 men and 40 women, who underwent recurrence-free survival for pancreatic cancer were included in this study. Table 1 summarizes the patient characteristics. The mean tumor diameter was 2.6 (range, 0.5–6.0) cm. The mean duration of surgery was 513 (range, 321–892) min. The mean blood loss was 788 (range, 17–8,220) mL. Eleven (13.8%) patients underwent blood transfusions. Postoperative complications of Clavien–Dindo class ≥3a were observed in 19 (23.8%) patients.

**Table 1:** Demographic and clinical characteristics of the patients with pancreatic cancer.

Variables	Total cases (n = 80) Mean ± SD or rate	Range
Age (years)	71.4 ± 8.5	46–88
Sex (male/female)	40/40	
BMI (kg/m^2^)	22.6 ± 3.8	15.4–33.2
CEA (ng/mL)	6.43 ± 18.06	0.5–162
CA19-9 (U/mL)	718.23 ± 1951.88	2–12000
Serum albumin level (g/dL)	3.8 ± 0.5	2.5–5.0
Preoperative inflammation-based markers		
CAR	0.23 ± 0.66	0.002–4.81
PLR	161 ± 76	51–428
NLR	2.92 ± 1.87	0.78–10.75
PNI	45.8 ± 6.36	28.3–61.0
PMI Male Female	6.76 ± 1.57 4.89 ± 1.27	3.85–10.37 2.59–7.67
Neoadjuvant chemotherapy; n (%)	28 (35.0%)	
Surgical procedure PD Subtotal stomach-preserving PD Vascular resection	6 (7.5%) 74 (92.5%) 30 (37.5%)	
Resectability Resectable Borderline resectable BR-PV BR-A	54 (67.5%) 25 (31.2%) 1(1.2%)	
Duration of operation (min)	513 ± 104	321–892
Blood loss (mL)	788 ± 1184	17–8220
Blood transfusion	11 (13.8%)	
Stage UICC eighth edition (IA/IB/IIA/IIB/IV)	8/5/29/33/5	
Tumor size (cm)	2.6 ± 1.0	0.5–6.0
Vascular invasion	26 (33.3%)	
Tumor differentiation Well/moderately/poor/others	46 (57.5%)/27 (33.8%) /3 (3.8%) /4 (5%)	
Mortality	1 (1.3%)	
POPF Grade ≥B n (%)	13 (16.2%)	
Postoperative complication (Clavien–Dindo ≥3a)	19 (23.8%)	
Adjuvant chemotherapy; n (%)	64 (84.2%)	
Resection curability; R0 n (%)	73 (91.2)	
Postoperative hospital stay (days)	26.5± 20.2	9–122

CAR, C-reactive protein-to-albumin ratio; CEA, carcinoembryonic antigen; CA19-9, cancer antigen 19–9; PLR, platelet-lymphocyte ratio; NLR, neutrophil-lymphocyte ratio; PNI, prognostic nutritional index; PMI, psoas muscle index; PD, pancreaticoduodenectomy; UICC, Union for International Cancer Control; POPF, postoperative pancreatic fistula; SD, standard deviation.

### ROC curve analysis of systemic inflammation-based biomarkers

ROC curve analysis was used to determine the optimal cut-off value for each systemic inflammation-based biomarker, with overall survival as an endpoint. The AUC values for CAR, neutrophil-lymphocyte ratio, platelet-lymphocyte ratio, and prognostic nutritional index were 0.67, 0.61, 0.57, and 0.66, respectively. The ability of CAR to predict overall survival in patients with pancreatic cancer was superior to that of the other markers. The optimal cut-off value for CAR was 0.47 (sensitivity, 66.7%; specificity, 68.3%), and the patients were divided into high (≥0.47; n = 39) and low (<0.47; n = 41) CAR groups based on this cut-off value.

### Comparison between the sarcopenia and non-sarcopenia groups

The cut-off values for PMI were 5.50 and 4.49 cm^2^/m^2^ in men and women (AUC: 0.55 and 0.71), respectively. Twenty-four (30.0%) patients were diagnosed with sarcopenia. The patients were stratified into two groups based on the presence (*n* = 24) or absence (*n* = 56) of sarcopenia. Table 2 presents the results of the comparison between the clinicopathological variables of the two groups. No significant differences were observed between the groups in terms of age or body mass index. The frequency of lymph node metastasis was higher in the sarcopenia group than in the non-sarcopenia group. The proportion of patients with stage UICC ≥ IIB in the sarcopenia group was higher than that in the non-sarcopenia group. A significant correlation was observed between sarcopenia and the progression of cancer.

**Table 2:** Clinicopathological features of the patients with pancreatic cancer according to sarcopenia status.

Variables	Sarcopenia group (n = 24)	Non-sarcopenia group (n = 56)	*p* value
Age (years) ≥75	9 (37.5%)	21 (37.5%)	1.000
Sex (male)	8(33.3%)	32 (57.1%)	0.050
BMI ≥25 (kg/m^2^)	6 (25.0%)	14 (25.0%)	1.000
CEA ≥7.5 (ng/mL)	6 (25.0%)	7 (12.5%)	0.178
CA19-9 ≥130 (U/mL)	11 (45.8%)	21 (37.5%)	0.487
Albumin ≥3.5 (g/dL)	17 (70.8%)	45 (80.4%)	0.358
Preoperative inflammation-based markers			
PLR ≥138	14 (58.3%)	32 (57.1%)	0.921
NLR ≥2.42	12 (50.0%)	29 (51.8%)	0.884
PNI ≥45.0	12 (50.0%)	34 (60.7%)	0.375
CAR ≥0.47	12 (50.0%)	27 (48.2%)	0.884
Neoadjuvant chemotherapy	8 (33.3%)	20 (35.7%	0.838
Resectability R	18 (75.0%)	36 (64.2%)	0.520
Vascular resection	12 (50.0%)	18 (32.1%)	0.134
Duration of operation ≥500 (min)	11 (45.8%)	31 (55.4%)	0.434
Blood loss ≥700 (mL)	10 (41.6%)	32 (57.1%)	0.204
Blood transfusion	2 (8.3%)	9 (16.1%)	0.511
Tumor size ≥3 (cm)	9 (37.5%)	19 (33.9%)	0.760
Lymph node metastasis	16 (66.7%)	21 (37.5%)	0.016*
Vascular invasion	11 (45.8%)	15 (26.8%)	0.100
Stage UICC ≥IIB	16 (66.7%)	22 (39.3%)	0.024*
Well-differentiated	12 (50.0%)	34 (60.7%)	0.376
Postoperative complication (Clavien–Dindo ≥3a)	5 (20.8%)	14 (25.0%)	0.686
Adjuvant chemotherapy	19 (79.2%)	45 (86.6%)	0.422
Resection curability; R0	22 (91.7%)	53 (94.6%)	0.623

BMI, body mass index; PLR, platelet-lymphocyte ratio; CEA, carcinoembryonic antigen; CA19-9, cancer antigen 19–9; NLR, neutrophil-lymphocyte ratio; PNI, prognostic nutritional index; CAR, C-reactive protein-to-albumin ratio; PMI, psoas muscle index; UICC, Union for International Cancer Control; SD, standard deviation.

### Survival analysis

**Fig 2:**
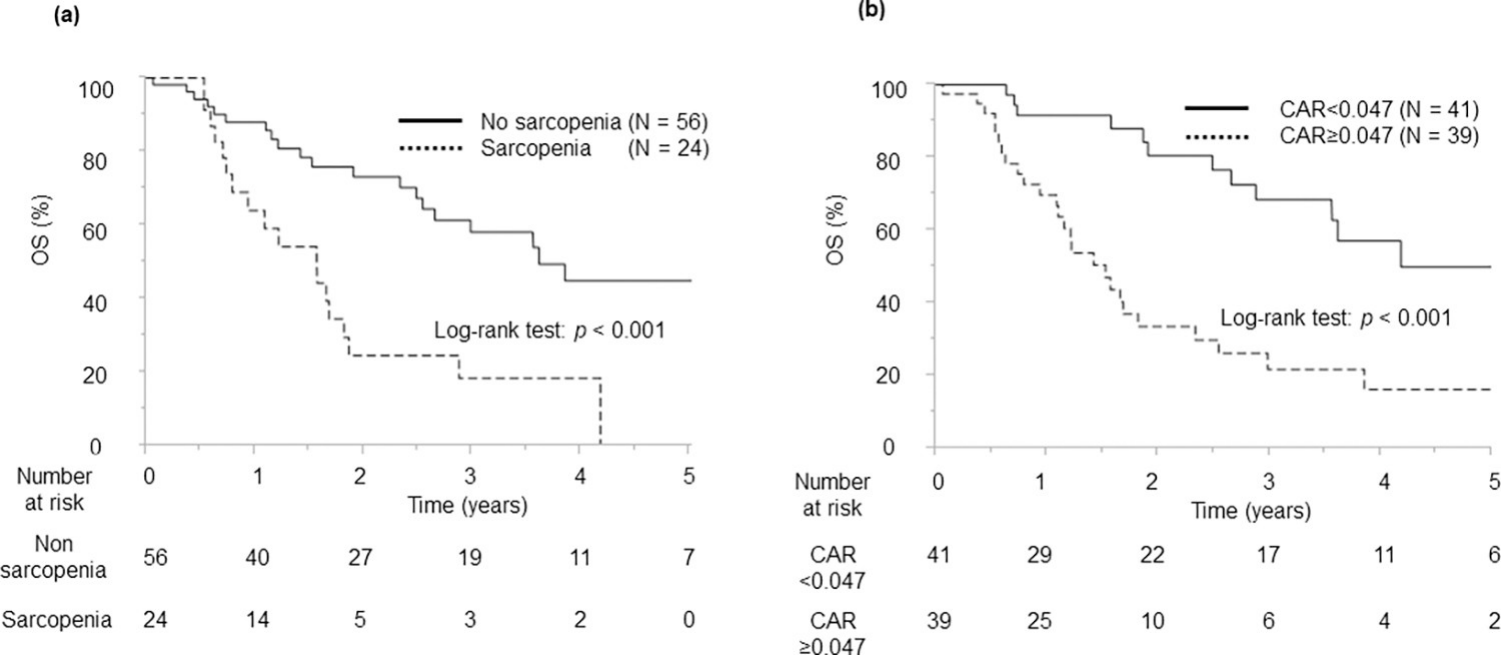
Kaplan–Meier analysis of overall survival. (a) The sarcopenia group versus the non-sarcopenia group. (b) The high CAR group versus the low CAR group. CAR, C-reactive protein-to-albumin ratio.

The median overall survival was 32.3 (range, 2.0–102.7) months, whereas the 1-, 3-, and 5-year overall survival rates were 89.4%, 44.8%, and 32.8%, respectively. Kaplan–Meier analysis revealed that the overall survival of the sarcopenia group was significantly shorter than that of the non-sarcopenia group (*P* < 0.001; Fig 2A). The overall survival of the high CAR group was significantly shorter than that of the low CAR group (*P* <0.001; Fig 2B). The median recurrence-free survival was 17.6 (range, 2.0–102.7) months, whereas the 1-, 3-, and 5-year recurrence-free survival rates were 53.9%, 42.3%, and 33.9%, respectively.

### Univariate and multivariate analyses of the clinicopathological characteristics in relation to overall survival and recurrence-free survival

Table 3 presents the relationship between clinicopathological characteristics and overall survival in patients with pancreatic cancer after pancreaticoduodenectomy. Univariate analysis revealed a significant reduction in the overall survival of patients with a prognostic nutritional index of <45, CAR of ≥0.047, CA19-9 levels of ≥130 U/mL, sarcopenia, lymph node metastasis, and vascular invasion. Multivariate analysis revealed that a CAR of ≥0.047 (hazards ratio [HR], 3.383; 95% CI: 1.384–8.689; *p*< 0.001), CA19-9 levels of ≥130 U/mL (HR, 2.720; 95% CI: 1.291–6.060; *p* = 0.008), sarcopenia (HR, 3.256; 95% CI: 1.535–7.072; *p* = 0.002), and vascular invasion (HR, 2.092; 95% CI: 1.057–4.170; *p* = 0.034) were independent predictors of overall survival. Table 4 presents the correlation between the clinicopathological characteristics and recurrence-free survival in patients with pancreatic cancer after pancreaticoduodenectomy. Univariate analysis revealed a significant reduction in the recurrence-free survival in patients with a CAR of ≥0.047, CA19-9 levels of ≥130 U/mL, sarcopenia, lymph node metastasis, vascular invasion, and tumor differentiation (no well-differentiated adenocarcinoma). Multivariate analysis revealed that CA19-9 ≥130 U/mL (HR, 3.177; 95% CI: 1.612–6.431; *p*< 0.001), sarcopenia (HR, 2.463; 95% CI: 1.208–5.012; *p* = 0.013) vascular invasion (HR, 2.133; 95% CI: 1.039–4.433; *p* = 0.039) and tumor differentiation (no well-differentiated adenocarcinoma) (HR, 2.90; 95% CI: 1.491–5.718; *p* = 0.002) were independent predictors of recurrence-free survival.

**Table 3:** Univariate and multivariate analyses of clinicopathological variables related to overall survival of patients with pancreatic cancer.

Variables	Univariate analysis		Multivariate analysis	
	N	*p*-Value	HR (95% CI)	*p*-Value
Age (years) ≥75 <75	30 50	0.636		
Sex Male Female	40 40	0.105		
BMI (kg/m^2^) ≥25 <25	20 60	0.463		
CEA (ng/mL) ≥7.5 <7.5	13 67	0.159		
CA19-9 (U/mL) ≥130 <130	32 48	<0.001*	2.720 (1.291–6.060)	0.008*
Albumin (g/dL) ≥3.5 <3.5	62 18	0.104		
PLR ≥138 <138	46 34	0.189		
NLR ≥2.42 <2.42	41 39	0.340		
PNI ≥45 <45	46 34	0.013*	1.339 (0.561–3.334)	0.513
CAR ≥0.047 <0.047	39 41	<0.001*	3.383 (1.384–8.689)	<0.001*
Sarcopenia None Present	56 24	<0.001*	3.256 (1.535–7.072)	0.002*
Duration of operation (min) ≥500 <500	42 38	0.233		
Blood loss (mL) ≥500 <500	38 48	0.329		
Lymph node metastasis None Present	43 37	<0.001*	1.774 (0.756–4.157)	0.185
Vascular invasion None Present	51 26	<0.001*	2.092 (1.057–4.170)	0.034*
Tumor differentiation Well Others	46 347	0.062		
Postoperative complication (Clavien–Dindo ≥3) None Present	61 19	0.109		

HR, hazard ratio; CI, confidence interval; BMI, body mass index; CEA, carcinoembryonic antigen; CA19-9, cancer antigen 19–9; PLR, platelet-to-lymphocyte ratio; NLR, neutrophil-to-lymphocyte ratio; PNI, prognostic nutritional index; CAR, C-reactive protein-to-albumin ratio.

* statistically significant.

**Table 4:** Univariate and multivariate analyses of the clinicopathological variables related to the recurrence-free survival of patients with pancreatic cancer.

Variables	Univariate analysis		Multivariate analysis	
	N	*p*-Value	HR (95% CI)	*p*-Value
Age (years) ≥75 <75	30 50	0.380		
Sex Male Female	40 40	0.260		
BMI (kg/m^2^) ≥25 <25	20 60	0.653		
CEA (ng/mL) ≥7.5 <7.5	13 67	0.979		
CA19-9 (U/mL) ≥130 <130	32 48	<0.001*	3.177 (1.612–6.431)	<0.001*
PLR ≥138 <138	46 34	0.178		
NLR ≥2.42 <2.42	41 39	0.098		
PNI ≥45 <45	46 34	0.131		
CAR ≥0.047 <0.047	39 41	0.007*	1.903 (0.969–3.823)	0.062
Sarcopenia None Present	56 24	0.004*	2.463 (1.208–5.012)	0.013*
Duration of operation (min) ≥500 <500	42 38	0.067		
Blood loss (mL) ≥500 <500	38 48	0.580		
Lymph node metastasis None Present	43 37	<0.001*	1.683 (0.760–3.718)	0.197
Vascular invasion None Present	51 26	<0.001*	2.133 (1.039–4.433)	0.039*
Tumor differentiation Well Others	46 347	0.005*	2.90 (1.491–5.718)	0.002*
Postoperative complication (Clavien–Dindo ≥3) None Present	61 19	0.239		

HR, hazard ratio; CI, confidence interval; BMI, body mass index; CEA, carcinoembryonic antigen; CA19-9, cancer antigen 19–9; PLR, platelet-to-lymphocyte ratio; NLR, neutrophil-to-lymphocyte ratio; PNI, prognostic nutritional index; CAR, C-reactive protein-to-albumin ratio.

* statistically significant.

### Effect of “sarcopenia and CAR status” on survival

**Fig 3:**
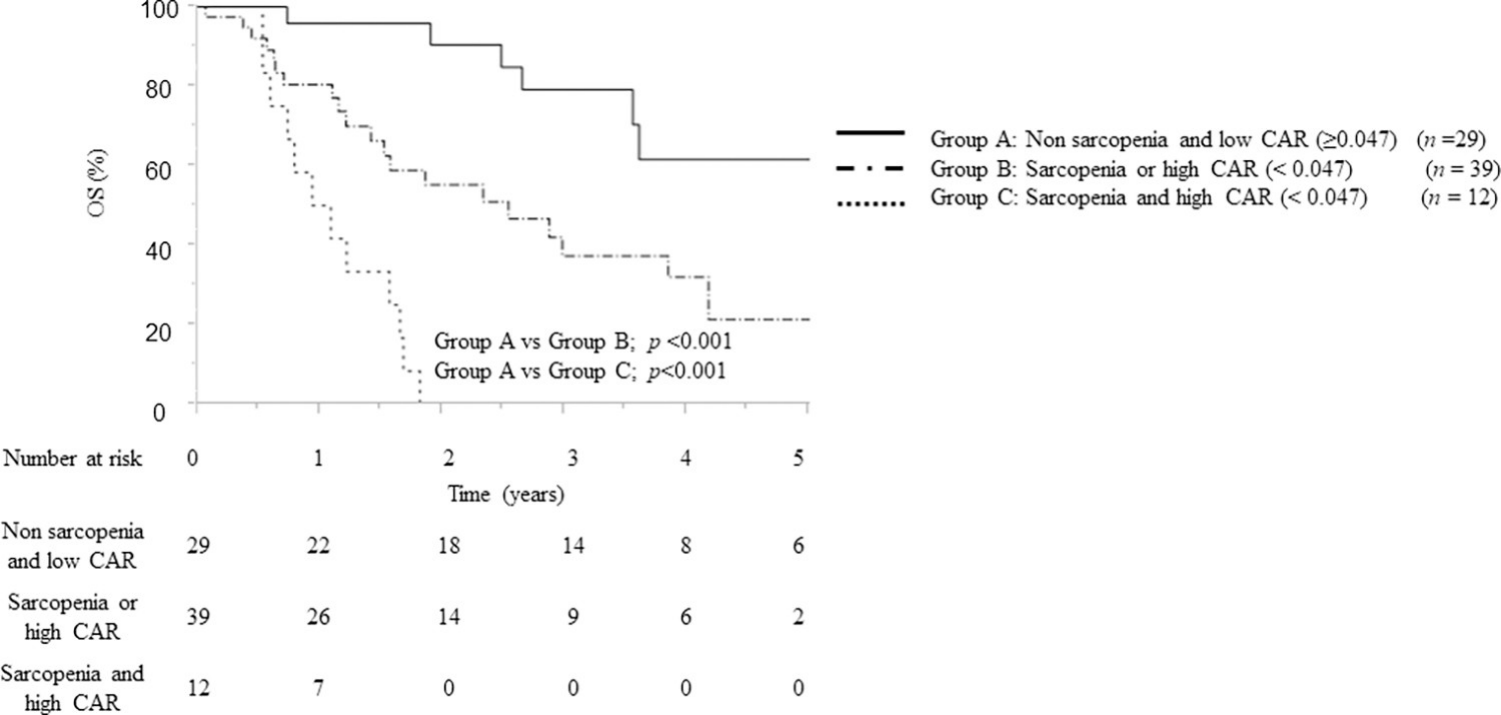
Kaplan–Meier curves according to sarcopenia and CAR status. CAR, C-reactive protein-to-albumin ratio.

Sarcopenia and high CAR were identified as significant prognostic factors for overall survival in patients with pancreatic cancer after pancreaticoduodenectomy. These two factors were combined to derive “sarcopenia and CAR status.” Overall survival was compared among the “non-sarcopenia and a low CAR (Group A),” “sarcopenia or a high CAR (Group B),” and “sarcopenia and a high CAR (Group C)” groups. The Kaplan–Meier curves were divided into three groups based on these statuses (Fig 3). Multivariate analysis was performed using the variable “sarcopenia and CAR status,” as sarcopenia and a high CAR showed a strong association with overall survival. This analysis revealed that “sarcopenia and a high CAR” and “sarcopenia or a high CAR” were significant prognostic factors for overall survival (HR: 14.393, 95% CI: 5.244–43.322, *P*<0.001; HR: 3.366, 95% CI: 1.469–8.703, *P* = 0.004, respectively) (Table 5).

**Table 5:** Multivariate analyses of overall survival including sarcopenia and CAR status.

Variables	Multivariate analysis	
	HR (95% CI)	*p*-Value
Sarcopenia and CAR status Non-sarcopenia and a low CAR Sarcopenia or a high CAR Sarcopenia and a high CAR	Ref. 3.366 (1.469–8.703) 14.393 (5.244–43.322)	0.004* <0.001*

CAR, C-reactive protein-to-albumin ratio; HR, hazard ratio; CI, confidence interval.

* statistically significant.

## Discussion

The present study demonstrated that sarcopenia and high CAR are independent predictors of poor overall survival in patients with pancreatic cancer and that the overall survival of the patients in the sarcopenia and high CAR group was the poorest among all three groups. The combination of sarcopenia and high CAR had a strong effect on poor survival. To the best of our knowledge, this study is the first to demonstrate that sarcopenia accompanied by CAR is correlated with poor prognosis in patients with pancreatic cancer after pancreaticoduodenectomy.

Sarcopenia is a hallmark of cancer cachexia. Moreover, it is a major factor that results in increased morbidity and mortality in patients with advanced gastrointestinal cancer [39,40]. The use of skeletal muscle index, which was calculated using the total muscle area at the L3 level, has been recommended by an international consensus for the evaluation of sarcopenia [41]. The PMI calculated by measuring the area of the psoas muscle at the L3 level showed a significant correlation with the skeletal muscle index according to the total muscle area. PMI has often been used as a marker for sarcopenia in patients with hepatobiliary cancer [8,9]. PMI can be calculated easily without the use of a special image-analysis system. Therefore, PMI was calculated to analyze sarcopenia in the present study.

Sarcopenia was identified as an independent prognostic factor for overall survival and recurrence-free survival in patients with pancreatic cancer after pancreaticoduodenectomy in the present study, which is consistent with the findings of previous studies [9,10]. A meta-analysis of prognostic factors for pancreatic cancer also demonstrated that sarcopenia is associated with poor survival in patients with pancreatic cancer [42]. Kim et al. revealed that muscle types with myosteatosis, regardless of the sarcopenia, were linked to poor overall survival in patients with pancreatic cancer. This result could be attributed to the different mechanism of sarcopenia and myosteatosis that contribute to nutritional and immunologic disturbances.

The present study revealed an association between sarcopenia and advanced tumor stage, suggesting that tumor progression is associated with sarcopenia. Previous studies have reported that inflammatory, immunological, and nutritional conditions are highly involved in carcinogenesis [43]. Various biomarkers that indicate the nutritional or inflammation level have attracted attention as potential biomarkers for predicting the prognosis of cancer. Some reports on preoperative inflammation-based biomarkers that assessed the prognosis of patients with pancreatic cancer have demonstrated that CAR may be useful in predicting prognosis [16,44]. A recent meta-analysis also revealed that CAR shows a significant association with poorer overall survival [45].

The levels of CRP, an acute-phase protein synthesized by the liver, increase rapidly in response to inflammation in patients with cancer [46]. Elevated CRP levels are associated with poor prognosis in various types of cancer [47,48]. Albumin, a protein produced by the liver, regulates osmotic pressure and functions as a carrier for the transportation of several metabolic substances. Hypoalbuminemia (malnutrition) is associated with poor overall survival in some types of cancer [49,50]. This finding may be attributed to several reasons. Albumin is an antioxidant that buffers biochemical changes, stabilizes cell growth and DNA replication, and maintains hormone homeostasis [51]. Moreover, high serum albumin levels may provide an antiproliferative effect in cancer cells *in vitro* [52]. Hypoalbuminemia is a syndrome associated with malnutrition and chronic inflammation [53]. Low serum albumin levels may weaken the immune system. This increases the susceptibility to infection, resulting in cytokine-induced suppression, which affects long-term overall survival [54]. Thus, CAR, which combines CRP and Alb, may be a prognostic indicator for patients with cancer, and high CAR levels could be a marker of poor overall survival.

Our study revealed that the sarcopenia and CAR strongly were correlated with poor prognosis. Rapid disease progression and poorer prognosis are observed in patients with sarcopenia accompanied by inflammation. The correlation between sarcopenia and systemic inflammatory markers has been attracting attention in recent years [25]. A strong correlation has been observed between systemic inflammatory markers and catabolic pathway activation [55]. Tumor necrosis factor and interleukin-6 released by the tumor and surrounding cells can suppress protein synthesis and stimulate protein degradation [56]. Tumors themselves also promote inflammation, thereby facilitating tumor progression. Secretion of pro-inflammatory myokines induces muscle degeneration and exacerbates systemic inflammation [57]. Inflammation may cause malnutrition, resulting in impaired immune responses and reduced muscle strength [58]. Thus, inflammation and malnutrition collectively lead to sarcopenia [59].

Appropriate preoperative nutritional therapy may improve the postoperative outcomes in patients with sarcopenia and a high CAR. Nakajima et al. [60] investigated the clinical benefits of preoperative exercise and nutritional therapy in patients undergoing hepatopancreatobiliary surgery for malignancies. Kaido et al. [8] reported the effects of nutritional therapy on the prognosis of patients with sarcopenia after liver transplantation. Preoperative rehabilitation is effective in reducing postoperative complications in patients with various types of cancer [61,62]. Thus, supportive therapies focusing on nutrition and rehabilitation should be implemented during the perioperative period in patients with pancreatic cancer. Especially, for patients with sarcopenia and high CAR, these supportive therapies might be expected to improve their prognosis. However, their efficacy must be evaluated in further prospective studies.

Preoperative PMI and CAR are reliable parameters for predicting the postoperative prognosis. Risk stratification for sarcopenia and inflammation-based biomarkers, which can be performed easily preoperatively, could aid clinical decision-making. However, further investigations are warranted when considering the indications for surgery in high-risk patients.

This study has certain limitations. First, this was a retrospective study with a small sample size. Second, since this was a retrospective study, there is a possibility of confounding bias. Third, the diagnostic criteria for sarcopenia were determined using only PMI. Sarcopenia should be diagnosed via the detection of low muscle mass and reduced muscle function. However, the data from physical tests such as the handgrip test, which indicates the degree of skeletal muscle function and is one of the diagnostic criteria for sarcopenia, was not available. It may be more suitable to combine these data with PMI to predict the prognosis of patients with pancreatic cancer. Finally, the clinical materials analyzed in this study were solely from a single institution in Japan. To overcome these limitations, large-scale, multicenter, prospective studies that include diverse ethnic populations are needed to further confirm the clinical relevancy of preoperative PMI in patients with pancreatic cancer.

In conclusion, sarcopenia and high CAR were associated with poor overall survival in patients with pancreatic cancer after pancreaticoduodenectomy. Moreover, sarcopenia and CAR were identified as independent preoperative predictors of overall survival in patients with pancreatic cancer. Nutritional therapy and rehabilitation may increase the survival of cancer patients with sarcopenia and inflammation.

## Supporting information

S1 File (XLS)
